# The importance of timing of socioeconomic disadvantage throughout development for depressive symptoms and brain structure

**DOI:** 10.1016/j.dcn.2024.101449

**Published:** 2024-09-13

**Authors:** Lia Ferschmann, Håkon Grydeland, Niamh MacSweeney, Dani Beck, Marieke G.N. Bos, Linn B. Norbom, Eira R. Aksnes, Mona Bekkhus, Alexandra Havdahl, Eveline A. Crone, Tilmann von Soest, Christian K. Tamnes

**Affiliations:** a PROMENTA Research Center, Department of Psychology, University of Oslo, Oslo, Norway; b Center for Lifespan Changes in Brain and Cognition, Department of Psychology, University of Oslo, Oslo, Norway; c Division of Mental Health and Substance Abuse, Diakonhjemmet Hospital, Oslo, Norway; d Leiden Institute for Brain and Cognition, Leiden University, Leiden, the Netherlands; e Institute of Psychology, Leiden University, Leiden, the Netherlands, Leiden University, Leiden, the Netherlands; f Center for Genetic Epidemiology and Mental Health, Norwegian Institute of Public Health, Oslo, Norway; g Nic Waals Institute, Lovisenberg Diaconal Hospital, Oslo, Norway; h Erasmus School of Social and Behavioral Sciences, Erasmus University Rotterdam, Rotterdam, the Netherlands

**Keywords:** ALSPAC, Brain structure, Depression, Development, MRI, Socioeconomic status

## Abstract

Prior studies have reported associations between socioeconomic disadvantage, brain structure and mental health outcomes, but the timing of these relations is not well understood. Using prospective longitudinal data from the Avon Longitudinal Study of Parents and Children (ALSPAC), this preregistered study examined whether socioeconomic disadvantage related differentially to depressive symptoms (n=3012–3530) and cortical and subcortical structures (n=460–733) in emerging adults, depending on the timing of exposure to socioeconomic disadvantage. Family income in early childhood and own income measured concurrently were both significantly related to depressive symptoms in emerging adulthood. Similar results were observed for perceived financial strain. In contrast, only family income in early childhood was associated with brain structure in emerging adulthood, with positive associations with intracranial volume and total and regional cortical surface area. The findings suggest that both objective and subjective aspects of one’s financial standing throughout development relate to depressive symptoms in adulthood, but that specifically early life family income is related to brain structural features in emerging adulthood. This suggests that associations between socioeconomic disadvantage and brain structure originate early in neurodevelopment, highlighting the role of timing of socioeconomic disadvantage.

## 1 Introduction

Mental health problems pose a considerable burden of disease (Global Burden of Disease Collaborative Network, 2015), with depressive disorders standing for a substantial amount of years lost due to disability or premature mortality (Rehm and Shield, 2019). Identifying environmental factors and biological mechanisms that contribute to the risk of developing depressive disorders is therefore crucial for reducing personal and societal costs. Socioeconomic disadvantage has been proposed as one such environmental factor that has impact on depressive disorders (Ridley et al., 2020). Recent advances in developmental sciences emphasize that the timing of exposure to adversity, for instance socioeconomic disadvantage, likely has differential consequences in terms of mental health risk and underlying brain structure and function (Ho and King, 2021, Merz et al., 2019).

The link between concurrent socioeconomic disadvantage and depression is well established (For reviews see Patel et al., 2018; Ridley et al., 2020). Studies have shown that sudden decreases in income can bring about poorer mental health (Christian et al., 2019, Kuhn et al., 2009). Sudden increases of income, on the other hand, tend to produce improvements in mental health and well-being (Wolfe et al., 2012). Similar effects have also been shown in anti-poverty randomized controlled trials (Haushofer and Shapiro, 2018).

There is also much evidence for positive associations between exposure to socioeconomic disadvantage in childhood and depression in adolescence and adulthood (Angelini et al., 2019, Butler, 2014, Domènech-Abella et al., 2021, Gilman, 2002, Gilman et al., 2003, Morrissey and Kinderman, 2020, Power et al., 2007, Tani et al., 2016). Studies have shown that exposure to socioeconomic disadvantage at different times through development relates to depressive symptoms later in time, above and beyond concurrent socioeconomic status (Angelini et al., 2019, Butler, 2014, Gilman, 2002, Gilman et al., 2003, Morrissey and Kinderman, 2020, Power et al., 2007). Many of these studies, however, relied on retrospective accounts of the family’s socioeconomic standing, and are thus subject to recall bias (e.g. Morrissey and Kinderman, 2020; Tani et al., 2016). There are several reasons why exposure to socioeconomic disadvantage early in development might be particularly consequential for mental health outcomes. First, it is widely accepted that the brain undergoes substantial structural and functional changes across childhood and into young adulthood (Lebel and Deoni, 2018, Norbom et al., 2021, Rubia, 2013, Tamnes et al., 2017), with the most pronounced changes taking place in the first years of life (Gilmore et al., 2018). Second, distinct neurobiological mechanisms (e.g. synaptogenesis, synaptic pruning, myelination) govern the different stages of development (Ho and King, 2021). Third, brain plasticity, an organism’s ability to adapt structurally and functionally to accommodate environmental demands, varies across the lifespan, and earlier stages of development are generally characterized by higher plasticity (Lindenberger and Lövdén, 2019). As individuals transition to adulthood, processes that decrease plasticity intensify and set the stage for greater stability (Lindenberger and Lövdén, 2019). This dynamic process of plasticity is considered adaptive as it allows the organism to change on the basis of early experiences to fit into later environments, however, it also carries potential costs and vulnerabilities (Belsky and Pluess, 2013). Experience of early socioeconomic adversity may disrupt the cellular and molecular processes involved in the development of structural and functional brain networks implicated in affect and cognition (Malave et al., 2022), resulting in increased risk of psychopathology, including depressive disorders (Ho and King, 2021). These temporarily distant associations are consistent with the Developmental Origins of Health and Disease Hypothesis, which emphasizes the significance of early life experiences on adult disease status, and also extends to mental health conditions (Forsdahl, 1977, Gluckman et al., 2010). To conclude, while theoretical and empirical studies suggest that socioeconomic disadvantage at different times throughout lifespan can be of importance for depressive disorders, there are currently few studies that examine the potentially differential impact of socioeconomic disadvantage at different stages of life on depressive symptoms.

A recent systematic review of links between socioeconomic disadvantage (measured with income-needs-ratio, education and neighborhood disadvantage) and brain structure and function (Rakesh and Whittle, 2021) found modestly convergent results suggesting that socioeconomic disadvantage is linked to reduced gray matter volume, surface area and cortical thickness, with more consistent findings for surface area than cortical thickness. The most implicated regions are widespread frontal and temporal regions, and hippocampal, amygdala and striatal volumes. For surface area, effects of income seem to be mainly global rather than regional (Norbom et al., 2023). A recent multi-sample lifespan study (Walhovd et al., 2022) showed that both income and education were more strongly related to intracranial volume (ICV) than to gray matter volume controlled for ICV. The authors conclude that the associations between indices of socioeconomic status and brain metrics are primarily grounded in neurodevelopment because ICV changes relatively little after school-age or mid-adolescence, while gray matter volume continues to change throughout the lifespan.

While all these studies contribute with important knowledge about socioeconomic status – brain relations, they do not provide insights into the timing effects of socioeconomic disadvantage. A handful of recent, albeit small studies (n < 55), have examined the temporal specificity of associations between socioeconomic disadvantage and brain structure (Dufford et al., 2020, Dufford et al., 2021, Ramphal et al., 2021). For example Dufford and colleagues (2021) found prospective associations between socioeconomic disadvantage across childhood and cortical thickness and surface area in adulthood; but no associations between concurrent socioeconomic disadvantage and brain structure in adulthood. Another study with children showed that hippocampal and amygdala volumes were more strongly related to earlier income-to-needs ratio than to later income-to-need ratio (Ramphal et al., 2021), again demonstrating the importance of timing.

Studies have started to distinguish between objective and subjective measures of socioeconomic disadvantage (Ahlborg et al., 2017, Green et al., 2024, Piera Pi-Sunyer et al., 2023, Quon and McGrath, 2014). An example of a subjective measure is financial strain, defined as a perception of adequacy of covering one’s expenses with available financial assets (Ettman et al., 2023). Financial strain is considered a measure of psychosocial stress and worries about material resources (Ettman et al., 2023), and can be considered a relative measure which is influenced by perceptions of others’ wealth (Shippee et al., 2012). Thus, even though such subjective measures are related to availability of objective financial assets, they are not restricted to lower socioeconomic status (Dijkstra-Kersten et al., 2015). They provide insights into additional life circumstances such as ability to make use of other resources such as social support, social services or self-efficacy, or other differences related to unique life circumstances that pose different financial demands or different expectations (Prawitz et al., 2006, Shippee et al., 2012). They also reflect processes related to social comparison and relative economic deprivation; the experience of inferior resources when compared to peers (Fröjd et al., 2006). Individuals with the same income can therefore experience different levels of financial strain. Objective and subjective measures of socioeconomic disadvantage are thus considered related, but separate constructs (Ettman et al., 2023).

Income, an objective measure that captures key facets of one’s financial condition, has been extensively studied in relation to both depressive symptoms (e.g. Alloush, 2022; Sareen et al., 2011) and brain structure (e.g. Norbom et al., 2023; Rakesh et al., 2022; Tomasi and Volkow, 2021). Less is known regarding how subjective measures of socioeconomic disadvantage relate to depressive symptoms and brain structure. Existing body of research suggest that both objective and subjective measures of socioeconomic disadvantage relate to depressive symptoms ((Dijkstra-Kersten et al., 2015; Ettman et al., 2023; Green et al., 2024; Piera Pi-Sunyer et al., 2023; Quon and McGrath, 2014), but see Dunn et al. 2008). However, which type of measure of socioeconomic disadvantage constitute the best predictor of mental health is largely unexplored.

### 1.1 Novelty and aims

This preregistered study aimed to explore the effects of timing of socioeconomic disadvantage on depressive symptoms and brain structure. The strength of the current study was the possibility to explore these associations in a longitudinal sample spanning 25 years and using both objective and subjective measures of socioeconomic disadvantage. To the best of our knowledge, this was also the first study to explore the effects of timing on brain structure in a large sample.

**Fig. 1:**
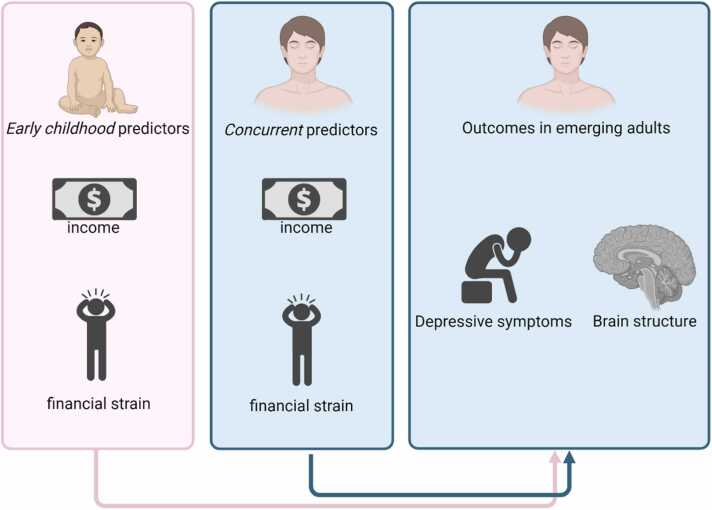
Illustrates the design of the present study where developmental origins effects of socioeconomic disadvantage (measured in early childhood) were compared to concurrent effects of socioeconomic disadvantage on depressive symptoms and brain structure in emerging adults.

The current study had three main objectives, all preregistered with their corresponding hypotheses. We aimed to test i) whether depressive symptoms in emerging adulthood relate to exposure to socioeconomic disadvantage in childhood above and beyond exposure to concurrent socioeconomic disadvantage (and vice versa), ii) whether brain structure relates to exposure to socioeconomic disadvantage in childhood and to concurrently experienced socioeconomic disadvantage and iii) explore whether the associations would differ with objective and subjective measures of socioeconomic disadvantage. We hypothesized that i) exposure to socioeconomic disadvantage both in childhood and concurrent exposure would be associated with depressive symptoms in emerging adulthood (Angelini et al., 2019, Butler, 2014, Christian et al., 2019, Gilman, 2002, Gilman et al., 2003, Morrissey and Kinderman, 2020, Power et al., 2007, Ridley et al., 2020, Tani et al., 2016), ii) adult brain structure would be more related to exposure to socioeconomic disadvantage in childhood than to concurrently experience socioeconomic disadvantage, with associations found particularly for ICV, SA, CT, as well as amygdala, hippocampal and striatal volumes (Dufford et al., 2021, Noble et al., 2015, Norbom et al., 2023, Rakesh and Whittle, 2021, Ramphal et al., 2021, Walhovd et al., 2022) and iii) stronger associations would be found for subjective measures of socioeconomic disadvantage because financial strain is also considered a measure of psychosocial stress related to life circumstances such as reduced access to social support, disproportionate spending habits or experience of relative economic deprivation (Fröjd et al., 2006, Prawitz et al., 2006, Shippee et al., 2012). The study’s design is illustrated in Fig. 1.

## 2 Methods

This study was preregistered on the Open Science Framework (osf.io/wnxd3). Any analytical deviations from the preregistrations are described below.

### 2.1 Sample

We used data from the Avon Longitudinal Study of Parents and Children (ALSPAC), a multigenerational prospective study where the initial data collection started with 14,541 pregnant women in 1990–92, and which has since collected data on the mothers, their children and other family members. Magnetic resonance imaging (MRI) data are available for a subsample (*n* = 961, aged 18–24) of ALSPAC participants from three different neuroimaging studies. Details about the study are provided elsewhere (Boyd et al., 2013, Fraser et al., 2013, Northstone et al., 2019, Sharp et al., 2020) and in the Supplementary Material. Please note that the study website contains details of all the data that is available through a fully searchable data dictionary and variable search tool (http://www.bristol.ac.uk/alspac/researchers/our-data/).

**Fig. 2:**
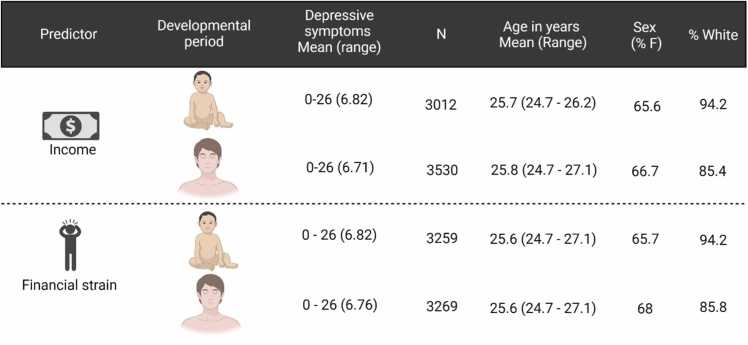
Information about the samples used in analyses exploring the associations between socioeconomic disadvantage at different developmental stages and depressive symptoms in young adulthood. Childhood family income and financial strain were reported at ages 2.6. Concurrent income and financial strain were reported at age 25 and 24, respectively. Age refers to time when depressive symptoms were measured.

**Fig. 3:**
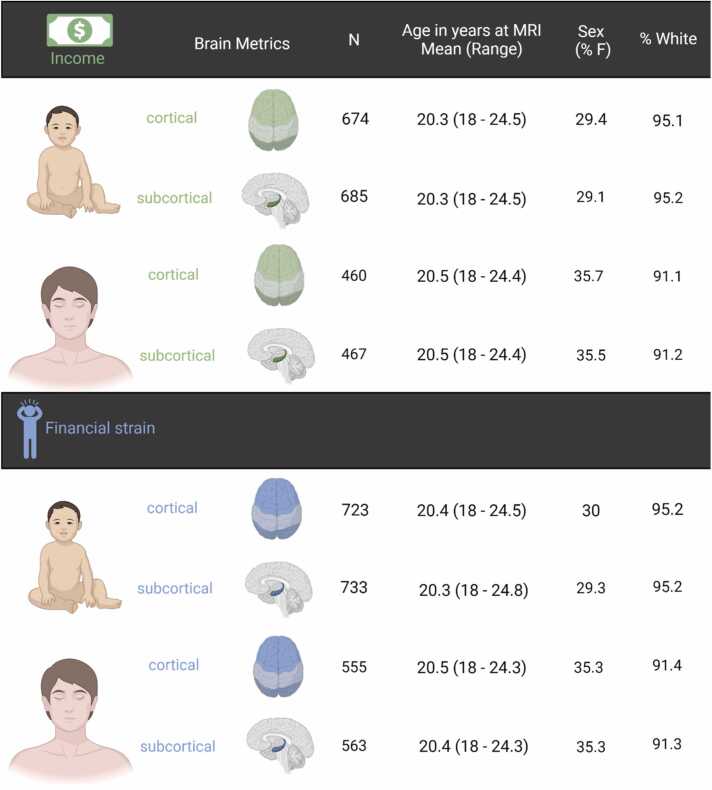
Information about the samples used in analyses exploring the associations between socioeconomic disadvantage at different developmental stages and cortical and subcortical brain structure. Childhood family income and financial strain were reported at ages 2.6. Concurrent income and financial strain were reported at age 25 and 24, respectively.

In analyses of brain structure, we only included participants with neuroimaging data that passed quality control (see below). Some individuals participated in more than one neuroimaging sub-study. If a person participated in more than one study, the data with the best quality, as indicated by the quality check variables provided by the ALSPAC study team, was included. If the quality was equal, data from one of the studies was randomly chosen. For each analysis, we required that the participants had complete data on both the outcome and the main predictor, see Fig. 2, Fig. 3 and Supplementary Material for more details. It should be noted that some of the participants with available neuroimaging data (n=126) had at least one definite or suspected psychotic experience using the psychotic-like symptoms semi-structured interview (PLIKS; Horwood et al., 2008) and 98 participants had high genetic risk for schizophrenia (see Supporting Materials on pages 1–2 and Sharp et al. 2020 for details). These participants were removed in sensitivity analyses as preregistered.

### 2.2 Measures

#### 2.2.1 Socioeconomic disadvantage

Socioeconomic disadvantage was measured with income and perceived financial strain (thereafter referred to as financial strain) from time points informed by developmental literature and data availability. We used data from age 2.6 years, the only time in early childhood when both measures of socioeconomic disadvantage were available. Concurrent measures of income and financial strain were collected at ages 25 and 24, respectively. These time points were chosen to keep them close in time to the available neuroimaging data, while also targeting a life period when most individuals likely have finished their higher education and have become relatively economically independent.

Income at age 2.6 years was reported by mothers as family weekly income in British pounds and was classified into 5 bands: <100, 100–199, 200–299, 300–399, >400). Income at age 25 years was reported by the respondents as monthly income in British pounds and was classified into 8 bands: 1–199, 200–299, 300–399, 400–599, 600–899, 900–1149, 1150–1499, 1500+ (Table 1).

**Table 1:** shows distributions of the different measures of socioeconomic disadvantage.

Measure	Age	Mean (SD)	Range
Income	2.6	3.57 (1.19)	1–5
	24	3.49 (1.24)	1–7
Financial strain	2.6	0.38 (1.03)	0–4
	25	0.3 (0.93)	0–4

Financial strain at ages 2.6 years and 24 years were assessed using an item from the Life Event Questionnaire (Rai et al., 2012). Respondents (mothers when the children were 2.6 years and the children themselves when they were 24 years) were asked whether they had major financial problems in the past year/since the last assessment and the degree to which they were affected by these financial difficulties. The responses were coded as 0 (did not happen), 1 (yes, but did not affect me), 2 (yes, mildly affected), 3 (yes, moderately affected), 4 (yes and affected me a lot). See Table 1.

#### 2.2.2 Depressive symptoms

**Fig. 4:**
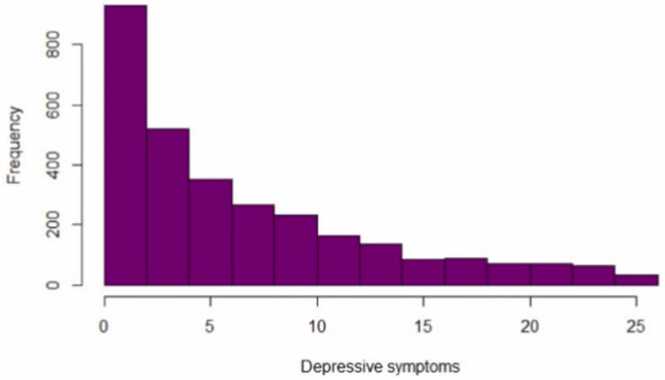
Distribution of depressive symptoms in emerging adulthood.

Depressive symptoms at 25 years were measured using the 13-item short Moods and Feelings Questionnaire (sMFQ; Angold and Costello, 2013), which assesses depressive symptoms in the past 14 days (Fig. 4). The items were rated as 0 (not true), 1 (sometimes), and 2 (true). The total score could range between 0 and 26. In this study, data collected at age 25 years were used in the analyses, i.e., a similar age at which concurrent measures of income and financial strain were collected. The Cronbach’s alpha was .92. suggesting that the scale had a good internal consistency.

#### 2.2.3 Brain Structure

Magnetic resonance imaging (MRI) data were collected at Cardiff University Brain Research Imaging Centre (CUBRIC) on a 3-T General Electric HDx using an 8-channel head coil. T1-weighed images were acquired using a FSPGR sequence. For the preregistered analyses, we used tabulated MRI-data processed and quality-checked by the ALSPAC team, more details in Sharp et al. (2020). Cortical reconstruction and volumetric segmentation was performed with FreeSurfer version 6.0.0 (Fischl, 2012). Specifically, we used following measures: cortical thickness of the left hemisphere, cortical thickness of the right hemisphere, surface area of the left hemisphere, surface area of the right hemisphere, total intracranial volume, volume of the left putamen, volume of the right putamen, volume of the left caudate, volume of the right caudate, volume of the left hippocampus, volume of the right hippocampus, volume of the left amygdala, volume of the right amygdala. Total cortical thickness and total surface area were calculated by adding values from left and right hemispheres. Similarly, hippocampal and amygdala volumes were created by summing values across hemispheres. Striatal volumes were calculated by adding caudate and putamen values from both hemispheres. For the follow-up sensitivity analyses, FreeSurfer version 7.1.1a was used to create a surface map for surface area (SA) of the cerebral cortex. These SA surface maps were smoothed with a Gaussian kernel of full-width/half-maximum of 12 mm and submitted to vertex-wise group analyses.

#### 2.2.4 Covariates

Sex assigned at birth was indicated either as male or female. Child’s ethnicity was reported by the mothers as white and non-white. Although current guidelines do not recommend this distinction (Flanagin et al., 2021), we still used this variable as there was very little variation in the data on ethnicity and ethnicity was not the research focus in this paper. Maternal depressive symptoms at child’s age 21 months was assessed using the Edinburgh Postnatal Depression Scale (EPDS; Levis et al., 2020). It is a 10-item instrument with statements such as “I have been so unhappy that I have had difficulty sleeping”. In the ALSPAC study, the statements were rated on a scale from 1 to 4 with lower scores representing more depressive symptoms (except for items 1, 2 and 4 which were reverse-coded). Cronbach’s alpha for this scale was .87.

### 2.3 Statistical analyses

Statistical analyses were performed using R version 4.1.2 (R Core Team, 2021) in RStudio version 1.4.1717 (RStudio Team, 2020), and in FreeSurfer version 7.1.1 (https://surfer.nmr.mgh.harvard.edu/).

Prior to preregistration we conducted several minor analyses which are described here: osf.io/wnxd3. To assess stability over time, Pearson correlations were performed to assess the correlation between family income in childhood and concurrent income and between mother-reported financial strain in childhood and concurrent financial strain. In the preregistration we planned to only include individuals that had information on socioeconomic disadvantage from both childhood and emerging adulthood. However, preparing the dataset for analyses revealed that this strict inclusion criterium would mean losing up to nearly 300 participants (41.1 %) with MRI data (Supplementary Table 1). For that reason, we decided to instead include the maximal possible sample at each time point and for each measure. For transparency, we include analyses with the strict inclusion criterium in the Supplementary Materials. We also performed post-hoc Welch Two Sample t-tests to examine whether our sample differed in socioeconomic status and depressive symptoms in comparison to those who were recruited into the ALSPAC study but were not included in our analyses.

In our main analyses, we ran a series of generalized linear models (GLM) by means of the *glm* function in R. In analyses with depression score as an outcome variable, a Poisson distribution was chosen, because depression score is a count variable. However, since the assumption of no overdispersion was not met, a quasi-Poisson distribution was chosen instead. For the remaining analyses with brain structure as outcome variables, we chose a gaussian distribution. First, to test whether socioeconomic disadvantage relate to depression levels in emerging adulthood we ran four GLMs with depressive symptoms at age 25 as outcome and socioeconomic disadvantage (income age 2.6 / income age 24 / financial strain age 2.6 / financial strain age 25), age at depression measurement and sex as predictors. To correct for four comparisons, the Benjamini-Hochberg procedure to control for false discovery rate (FDR) was applied by means of the p.adjust () function in R.

In instances of significant associations, we ran additional sensitivity analyses by including ethnicity and maternal depression as additional covariates. For both income and financial strain, we also ran additional models where we investigated unique contributions of socioeconomic disadvantage at each time point by entering socioeconomic disadvantage from both timepoints simultaneously. Doing so gave us information about the association between depressive symptoms in emerging adulthood and childhood socioeconomic disadvantage above and beyond concurrent socioeconomic disadvantage, and vice versa.

Second, to test whether socioeconomic disadvantage relate to brain structure in emerging adults, we ran a series of GLMs with brain structure as outcome (total cortical thickness, total surface area, ICV, hippocampal, amygdala and striatal volumes) and socioeconomic disadvantage, age at MRI acquisition and sex as predictors. Total of 24 analyses. Again, we used the FDR procedure to correct for 24 comparisons. In instances of significant associations between socioeconomic disadvantage and subcortical structure, we ran additional sensitivity analyses with ICV as an additional covariate. Similarly, all significant associations between socioeconomic disadvantage and any brain modality were repeated while excluding any participants (n = 173) with psychotic experiences and high genetic risk for schizophrenia (see Sharp et al. 2020 for details). To examine how the association between childhood income and brain structure differs when concurrent income is included as an additional covariate, we conducted additional post-hoc analyses on a subset of individuals who had data on both childhood and concurrent income.

Third, standardized beta values were compared to assess whether income or financial strain had strongest links to depressive symptoms and brain structure in emerging adults. In post-hoc analyses, with the aim of better assessing the contribution of subjective as opposed to objective measures of socioeconomic disadvantages, we extended the analyses where depression was regressed on income with a measure of perceived financial strain from the same time point. One model included childhood income and childhood financial strain, and the other model included concurrent income and concurrent perceived financial strain.

Furthermore, we performed exploratory analyses to further probe the regional specificity of the association between childhood family income and total surface area. These analyses were not preregistered. While many studies find links between socioeconomic disadvantage with surface area (for review see Rakesh and Whittle, 2021), inconsistencies exist in terms of whether the effects are mainly global (e.g. Norbom et al., 2023) or local (e.g. Noble et al. 2015). Some of these inconsistencies can be traced to different methods and samples, but also to a tendency to choose specific, particularly frontal regions (Thomas and Coecke, 2023). We chose to perform additional exploratory analyses to provide more nuanced knowledge on the association between socioeconomic disadvantage and surface area. Specifically, we performed vertex-wise analyses using the mri_glmfit command in FreeSurfer. This involved fitting separate GLMs of the effects of childhood income on surface area at each vertex in each hemisphere, controlling for sex and age. Given the large number of vertices, we performed a clusterwise correction for multiple comparisons using Monte Carlo simulation to obtain a distribution of maximum cluster size under the null hypothesis (Hagler et al., 2006, Hayasaka, 2003). We used a stringent cluster-forming threshold of p <.001 (two-sided), in line with (Greve and Fischl, 2018), yielding clusters and corresponding cluster P values corrected for multiple comparisons. Cluster P value significance level was set at.025 (two-sided)*.* Finally, to visualize relations and perform sensitivity analyses, we extracted values from the significant clusters. For each cluster, we performed a GLM to test whether childhood income was related to SA even when including only healthy controls (i.e., excluding individuals with psychotic experiences and high genetic risk for schizophrenia).

In post-hoc analyses, we explored further associations between brain structure, depressive symptoms, and socioeconomic disadvantage. Specifically, the associations between depressive symptoms and those brain metrics that have been associated with socioeconomic disadvantage. First, we estimated correlations between measures of brain structure that had been significantly predicted by socioeconomic disadvantage and depressive symptoms. In the next step, we ran GLMs where we regressed these brain metrics on depressive symptoms while controlling for age and sex. Additionally, to investigate the extent to which maternal depression was associated with future depressive states of their children, a GLM was performed where adult depressive symptoms were regressed on maternal depression, age and sex assigned at birth. For this analysis, we included all individuals that had data on depressive symptoms and maternal EPDS.

## 3 Results

Analyses conducted prior to preregistration revealed weak but significant correlations between family income in childhood and concurrent income (*r* =.17, *p* <.001) and between mother-reported financial strain in childhood and concurrent financial strain (*r* =.10, *p* <.001).

**Fig. 5:**
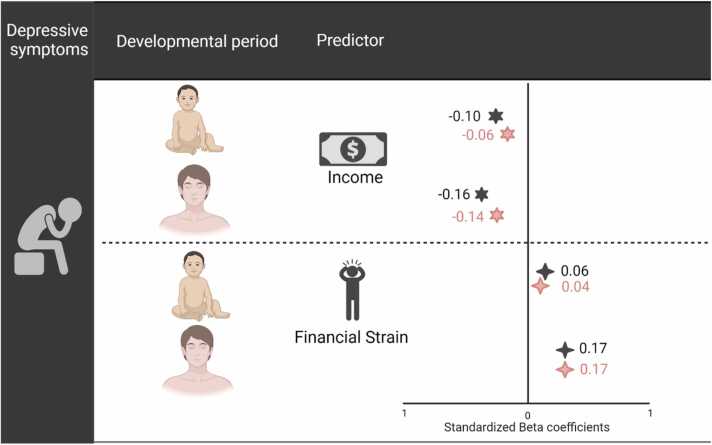
Variance in depressive symptoms in emerging adulthood explained by socioeconomic disadvantage at different developmental stages. Black stars = effects of socioeconomic disadvantage at that particular time on adult depression. Pink stars = effects of socioeconomic disadvantage at that particular time above and beyond socioeconomic disadvantage at the other time point.

Attrition analyses suggested that on average, participants in this study came from families with higher income at the child’s age of 2.6 years (*M* = 3.57, *SD* = 1.19) than those who were not included in this study (*M* = 3.22. *SD* = 1.27; *t*(8815)= −12.86, *p* < 0.001). Hedge’s *g* is an appropriate measure effect size with unequal sample sizes was calculated to be *g* = 0.27, suggesting a small-sized effect. Depressive symptoms did not differ significantly between groups (*t*(1594) = 1.93, *p* =.277). We first tested whether socioeconomic disadvantage at different developmental stages was associated with depressive symptoms in emerging adulthood, controlling for age and sex. Effect sizes are depicted in Fig. 5. Both childhood income and concurrent income were negatively associated with depressive symptoms in emerging adults (*p*_corrected_ <.0001, 95 % CI [-0.14,-0.07] and [-0.19,-0.12] respectively). The associations remained significant after controlling for ethnicity and maternal depression. When simultaneously entered into the model, both childhood income (*p*_corrected_ = 0.003) and concurrent income (*p*_corrected_ <.0001) uniquely explained variance in depressive (95 % CI [-0.09,-0.02] and [-0.18,-0.11] respectively). The unique effect of concurrent income, but not childhood income, remained significant even when additionally controlling for ethnicity and maternal depression. When controlling for sex, age, concurrent income, ethnicity and maternal depression, the β-value declined to −.05, *p*_uncorrected_ = 0.011.”

Similar patterns were found for financial strain. Both childhood and concurrent financial strain were positively associated with depressive symptoms in emerging adulthood (*p*_corrected_ <.0001, 95 % CI [0.03–0.09] and [0.14–0.20] respectively). The associations remained significant after controlling for ethnicity and maternal depression. When simultaneously entered into the model, both childhood financial strain (*p*_corrected_ = 0.042) and concurrent financial strain (*p*_corrected_ <.0001) uniquely explained variance in depressive symptoms (95 % CI [0.001–0.07] and [0.14–0.20] respectively). The unique effect of concurrent financial strain remained significant when additionally controlling for ethnicity and maternal depression ( *p*_corrected_ <.0001), while the unique effect of childhood financial strain was no longer significant (*p*_corrected_ = 0.066). In summary, our hypothesis that exposure to socioeconomic disadvantage both in childhood and concurrent exposure would be associated with depressive symptoms in emerging adulthood was largely supported.

Next, we tested whether socioeconomic disadvantage at different developmental stages predicted brain structure (total cortical thickness, total surface area, ICV, hippocampal, amygdala and striatal volumes) in emerging adulthood, controlling for age and sex. Childhood income was associated with total SA (β = 0.08, *p*_corrected_ = 0.049, 95 % CI [0.017–0.14]) and ICV (β = 0.09, *p*_corrected_ = 0.019, 95 % CI [0.03–0.15]). The results remained significant after exclusion of individuals with psychotic experiences and high genetic risk for schizophrenia. Post-hoc analyses examining how the association between childhood income and ICV/surface area would change if concurrent income was included was performed on a sample that had data on both childhood and concurrent income. The results suggested that beta values were only slightly changed when concurrent income was included, and the associations are therefore not driven by concurrent income. Details can be found in Supplementary tables 22–24. Childhood income was also positively associated with hippocampal and amygdala volumes, but not after including ICV as an additional covariate. No associations were found between childhood income and total cortical thickness or striatal volumes. No associations were found between concurrent income and any brain metrics. No associations between childhood or concurrent financial strain and brain structure in emerging adulthood were identified. In summary, our hypothesis that lower income and more financial strain in early childhood would be linked to reduced hippocampal, striatal and amygdala volumes, and lower total surface area was partially supported, with associations seen only between income and cortical surface area and ICV.

**Fig. 6:**
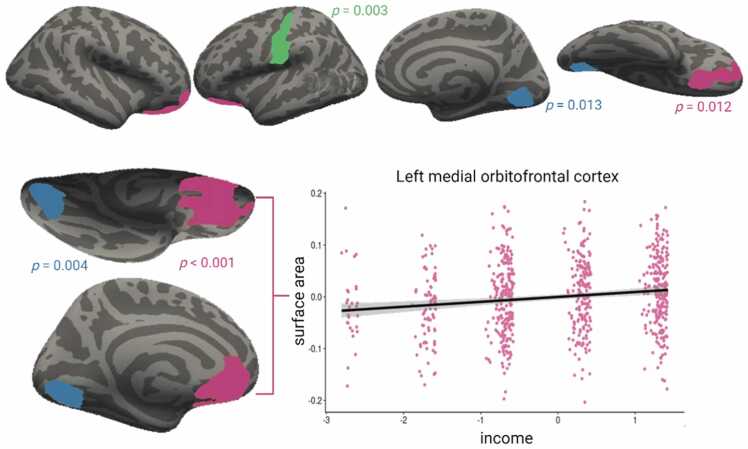
Vertex-wise analyses showed positive associations between childhood income and surface area, controlling for age and sex. The scatterplot shows residual surface area against income in the left orbitofrontal cluster. Scatterplots from other significant clusters showed similar associations (Supplementary Figure 7).

Follow-up exploratory vertex-wise analyses controlling for age and sex yielded significant associations between childhood income and cortical surface area in five clusters: bilaterally in the orbitofrontal cortices and lingual gyrus, and in the left postcentral gyrus (Fig. 6). Given the positive associations between childhood income and both total and regional surface area, we explored whether the associations were mainly specific to the identified clusters or, rather, more homogenous across the surface, with the clusters representing peaks due to the thresholding inherent in cluster-based corrections for multiple comparisons. To do so, we examined the uncorrected (for multiple comparisons) significant maps of the regional SA analysis, threshold at a more liberal threshold (*p* < 0.05). As shown in Supplementary Figure 8, the associations between total surface area and childhood income appeared to be mainly driven by surface area in the identified clusters. Finally, using values from the significant clusters, we examined the associations between family income in childhood and surface area after excluding individuals with psychotic experiences or high risk for schizophrenia. The associations remained significant in all clusters.

Finally, as preregistered, we examined whether associations between socioeconomic disadvantage and depressive symptoms and brain structure differed for objective (income) and subjective (financial strain) measures. With regard to depressive symptoms in emerging adults, comparisons of the sizes of the standardized *beta* coefficients suggested that the association between childhood income and depressive symptoms was stronger than for childhood financial strain, while concurrent financial strain were more strongly linked to depressive symptoms than concurrent income. Comparison of beta values from post-hoc analyses where both subjective and objective measures from the same time point where included in the same model in predicting depression suggested, in line with the preceding results, that childhood income was more strongly related to adult depression than childhood financial strain. In contrast to the previous analyses, however, both concurrent income and concurrent financial strain had nearly equivalent beta coefficients. More details are included in Supplementary Information on page 13. While associations were found between childhood family income and brain structure, no associations were identified between financial strain and brain structure. In summary, our hypothesis that associations between subjective socioeconomic disadvantage and depressive symptoms and brain structure would be stronger than those for objective socioeconomic disadvantage was not supported.

Post-hoc analyses probing associations between depressive symptoms, brain structure and socioeconomic disadvantage, which included brain structures that had been linked to socioeconomic disadvantage in this study, revealed that depressive symptoms were negatively correlated both with ICV (r =-0.12, p =.012) and total surface area (r = - 0.15, p =.002). However, neither ICV (β = −0.0000002, p =.642) or total surface area (β = −0.000005, p =.151) were associated with depressive symptoms in GLMs where depressive symptoms were regressed on brain structure, age and sex. Post-hoc analyses examining the extent of the association between maternal depression and children’s adult depressive symptoms revealed a positive association (β = 0.08, *p* <.001, 95 % CI [0.05–0.10]).

## 4 Discussion

Using prospective longitudinal data, this preregistered study revealed that exposure to socioeconomic disadvantage both in early childhood and concurrently experienced socioeconomic disadvantage in emerging adulthood were uniquely related to depressive symptoms in young adults. In contrast, family income in childhood, but not concurrent income, was positively associated with inercranial volume and total and regional cortical surface area in young adults, indicating that potential influences of socioeconomic disadvantage on brain structure originate in early neurodevelopment. No associations were found between adult depressive symptoms and either intercranial volume or total surface area. The study highlights the importance of timing for socioeconomic disadvantage – depressive symptoms relations, as well as socioeconomic disadvantage – brain structure relations, but did not provide evidence for brain structure as the underlying mechanism for the association between socioeconomic disadvantage and depressive symptoms.

Our study first explored the effects of timing of exposure to socioeconomic disadvantage on depressive symptoms in emerging adulthood by comparing the associations between socioeconomic disadvantage in early childhood and concurrently measured socioeconomic disadvantage and depressive symptoms in emerging adulthood. We found that both socioeconomic disadvantage in early development and in young adulthood were uniquely and positively associated with depressive symptoms in emerging adults.

Given that overwhelming majority of the study participants reported not having experienced any financial difficulties, our results suggest that the associations are not merely restricted to poverty, but rather that normal variation in income and financial strain are linked to depressive symptoms. This is in line with existing literature that finds dose-response associations between socioeconomic status and mental health in high-income and upper-middle-income countries (Ni et al., 2023).

Our results complement the existing body of research (Angelini et al., 2019, Butler, 2014, Gilman, 2002, Gilman et al., 2003, Morrissey and Kinderman, 2020, Power et al., 2007, Tani et al., 2016) that has demonstrated links between exposure to socioeconomic disadvantage earlier in development and depressive symptoms later in time (above and beyond concurrent socioeconomic status). Our results also extend the existing body of literature by using both objective and subjective measures of socioeconomic disadvantage and by overcoming some of the methodological challenges found in the other studies such as reliance of retrospective recall av socioeconomic disadvantage (Morrissey and Kinderman, 2020) or retrospective recalls of maternal depression (Butler, 2014). The finding that exposure to socioeconomic disadvantage in early childhood uniquely predicts depressive symptoms in emerging adulthood emphasizes the importance of securing economically optimal environment for developing children. Our results, however, also show that independent of family income in early childhood, one’s concurrent exposure to socioeconomic disadvantage is also linked to depressive symptoms. The findings that both family income in early childhood as well as concurrent socioeconomic disadvantage are uniquely associated with depressive symptoms, thus suggest an important role of individuals’ economic standing throughout development. These findings are also in line with a recent model of depression and anxiety that suggests causal pathways from socioeconomic disadvantage to depression and vice versa, and proposes mechanisms spanning temporarily from early development to concurrent effects (Ridley et al., 2020). For example, some of the temporarily distant causal pathways can be linked to early fetal and early life biological sensitivity to environmental factors, in line with the Developmental Origins of Health and Disease Hypothesis (Forsdahl, 1977, Gluckman et al., 2010), while the more immediate mechanisms linking socioeconomic disadvantage with poorer mental health can include worries, poorer physical health, unfavorable living conditions or greater risk of being exposed to traumatic events (Ridley et al., 2020). Consequently, the pathways from socioeconomic disadvantage to depressive disorders may differ in character depending on when in development the exposure takes place*.* It needs to be acknowledged, however, that when simultaneously controlling for age, sex, ethnicity, concurrent measure of socioeconomic disadvantage and maternal depression, neither childhood income nor childhood financial strain were significantly associated with adult depressive symptoms. In these analyses including multitude of covariates, maternal depression predicted adult depression from childhood income and from childhood financial strain (β =.08 and β =.010, respectively, both *p* <.001). Our post-hoc analyses also revealed a positive association between maternal depression and the participants’ depressive symptoms in emerging adulthood. This highlights the significance of maternal depression for the risk of developing depression in adult age. Indeed, much research finds that maternal depression in early infancy contributes to child risk of psychopathology through various biological and psychosocial pathways, and the associations may be amplified in the context of socioeconomic disadvantage (Goodman, 2020, Goodman et al., 2011, Sawyer et al., 2019). This indicates that children of depressed mothers may benefit particularly from efforts to alleviate socioeconomic disadvantage*.* Second, our study aimed to explore the effects of timing of exposure to socioeconomic disadvantage on brain structure in emerging adulthood. As expected based on prior studies (Brito and Noble, 2018, Noble et al., 2015, Walhovd et al., 2022), we found positive associations between income and total surface area and ICV, respectively. Since surface area and ICV have reached their maximum size by midadolescence (Amlien et al., 2016, Mills et al., 2016), this may imply that the relationship between income and brain structure originates in early neurodevelopment, as shown for several aspects of socioeconomic status (Walhovd et al., 2022). Exploratory analyses furthermore implicated regional orbitofrontal, lingual, and the left postcentral cortical area, largely overlapping with existing studies (Noble et al., 2015). Intriguingly, these regions also overlap with regions where adolescents with major depressive disorders show reductions in surface in comparison to healthy controls (Schmaal et al., 2017). Identifying a link between surface area in these brain regions with depressive symptoms in the current sample could indicate brain structure as potential explanatory mechanism for the association between childhood socioeconomic disadvantage and depressive symptoms. However, despite the association between childhood socioeconomic disadvantage and both i) depressive symptoms and ii) global / regional surface area and ICV, no link was identified between brain structure and depressive symptoms. Future studies should target brain structures in regions identified in previous studies as potential mediators of the relation between socioeconomic disadvantage and later depressive symptoms. It could be of interest to examine whether socioeconomic disadvantage in early development affects cortical surface area expansion or cortical thickness development in frontal, temporal and higher-order visual, motor and somatosensory areas (for the ENIGMA-Major Depressive Disorder Working Group, 2017, Toenders et al., 2019) and creates vulnerability for depressive disorders.

Contrary to expectations, associations between family income in childhood and amygdala and hippocampal volumes were no longer significant when ICV was controlled for. Some previous studies found links between socioeconomic disadvantage and subcortical volumes while controlling for whole-brain volumes instead of ICV (e.g. Hanson et al., 2011; Luby et al., 2013; Merz et al., 2018). In one study (Koyama et al., 2023), early poverty was prospectively associated with smaller amygdala volumes also after controlling for ICV, but these effects were driven by prenatal poverty. The same study showed no association between postnatal poverty (1–5 years) and hippocampal or amygdala volumes at age 10 years. Ramphal and colleagues (2021) on the other hand found a positive association between early income-to-need ratio and hippocampal and amygdala volumes at ages 7–9 years, while controlling for ICV. Future well-powered studies need to clarify the links between socioeconomic disadvantage and subcortical volumes at different time points throughout development.

In general, our results support the notion that the timing of socioeconomic experiences is of relevance for brain structure in emerging adulthood. While family income in early childhood was positively associated with surface area and ICV in emerging adulthood, no concurrent effects of income on brain structure were found. This is in line with other studies that also identified a timing effect whereby socioeconomic disadvantage exerted greatest impact on brain development at earlier developmental stage as opposed to later (Dufford et al., 2021, Koyama et al., 2023, Ramphal et al., 2021). While the current study as well as other studies (Dufford et al., 2021, Koyama et al., 2023, Ramphal et al., 2021, Walhovd et al., 2022) suggest that the relations between socioeconomic standing and brain structure may be grounded in early neurodevelopment, further studies are needed to delineate which brain structures are most impacted at which developmental stage (including prenatal periods), and map the underlying mechanisms and functional consequences.

Contrary to our expectations, the most robust associations between socioeconomic disadvantage and depressive symptoms and brain structure were identified for income rather than financial strain. This was at least in part likely due to the low variability in financial strain in this sample, where an overwhelming majority of study participants reported having no financial strain. Even though the majority of individuals reported not having experienced major financial strain, associations were found with depressive symptoms but not with brain structure. It is plausible financial strain may have a stronger influence on depressive symptoms than on brain structure. It is also possible that the difference is caused by the comparatively smaller sample size in the analyses where financial strain was entered as predictors of depressive symptoms (n ∼3250) than in the analyses where this variable was examined as predictor of brain structure (n ∼ 650). This study cannot conclude why perceived financial strain has shown association with depressive symptoms but not with the brain structures*.* Despite the lack of findings in our study, considering both objective and subjective aspects of an individual’s socioeconomic standing can have important implications for both mental health and behavior (Hertz-Palmor et al., 2021, Miller et al., 2024), and should be examined in future studies.

The current study had several methodological strengths, including longitudinal data spanning 25 years, availability of data from several developmental periods for the same individuals and availability of MRI-data for a sub-sample. However, the study’s results must be considered in the light of its limitations. First, there was little variation in financial strain. Future studies should employ more subtle measures of subjective economic stress. The fact that most respondents reported not having experienced any financial strain, and attrition analyses revealed that our sample has slightly higher childhood income than non-participants, indicates that this study cannot provide conclusions about severe poverty and mental health and brain structure. Second, even though having measures of both income and financial strain from two developmental periods for all individuals constitutes a strength, it is possible that they may impact development through different mechanisms. For example, experiencing financial strain by the child’s mother may translate to reduced quality of parenting for the child (Jackson et al., 2000), while financial strain in emerging adulthood may be the result of psychosocial stress related to relative deprivation and social comparison (Fröjd et al., 2006, Shippee et al., 2012). Third, the MRI subsample comes from three different neuroimaging studies with three different purposes. As a result, the MRI subsample has some peculiar characteristics, such as consisting of more males than females, and including individuals who have had psychotic like experiences or with high genetic risk for schizophrenia. Sex was however included as a covariate in all analyses and sensitivity analyses without individuals with clinical or genetic risk for psychosis were performed. Third, the available MRI-data are cross-sectional which puts limits on which socioeconomic disadvantage-brain associations can be explored. Future studies could combine prospective data on socioeconomic disadvantage with longitudinal assessments of brain development. Future research could use triangulation of different causally informative approaches to better understand the nature of the relations between socioeconomic disadvantage, mental health and brain structure (Lawlor et al., 2017, Munafò et al., 2021).

In conclusion, using prospective longitudinal data, the present study showed unique associations between socioeconomic disadvantage in both early childhood and in emerging adulthood and depressive symptoms in young adults, indicating that individuals’ financial standing across different stages of development is relevant for depressive symptoms. In contrast, the study showed that early childhood family income, but not concurrent income, was associated with intracranial volume and total and regional cortical surface area, implying a lasting impact of early life factors on structural brain development.

## Funding

This work was supported by the Research Council of Norway (#223273, #288083, #323951, #325415, # 274611) and the South-Eastern Norway Regional Health Authority (#2021070, #2023012, #500189, #2020022). The UK Medical Research Council and Wellcome (Grant ref: 217065/Z/19/Z) and the 10.13039/501100000883University of Bristol provide core support for ALSPAC. This publication is the work of the authors and Lia Ferschmann will serve as guarantors for the contents of this paper. A comprehensive list of grants funding (PDF, 330KB) is available on the ALSPAC website. This research was specifically funded by 10.13039/100004440Wellcome Trust (PI:Anita Thapar), Wellcome Trust and MRC (#102215/2/13/2).

## CRediT authorship contribution statement

**Dani Beck:** Writing – review & editing, Data curation, Conceptualization. **Niamh MacSweeney:** Writing – review & editing, Methodology, Conceptualization. **Linn B. Norbom:** Writing – review & editing, Conceptualization. **Marieke G.N. Bos:** Writing – review & editing, Conceptualization. **Christian K. Tamnes:** Writing – review & editing, Project administration, Methodology, Funding acquisition, Conceptualization. **Tilmann von Soest:** Writing – review & editing, Methodology, Conceptualization. **Håkon Grydeland:** Writing – review & editing, Methodology, Formal analysis, Conceptualization. **Lia Ferschmann:** Writing – review & editing, Writing – original draft, Visualization, Project administration, Methodology, Formal analysis, Conceptualization. **Eveline Crone:** Writing – review & editing, Conceptualization. **Alexandra Havdahl:** Writing – review & editing, Project administration, Conceptualization. **Eira R. Aksnes:** Writing – review & editing, Conceptualization.

## Declaration of Competing Interest

The authors declare that they have no known competing financial interests or personal relationships that could have appeared to influence the work reported in this paper.

## Data Availability

The data can be accessed pending an ethical approval, more details can be found here: https://www.bristol.ac.uk/alspac/researchers/access/

## Footnotes

Appendix A Supplementary data associated with this article can be found in the online version at doi:10.1016/j.dcn.2024.101449.
