# Supplementary material for: The importance of timing of socioeconomic disadvantage throughout development for depressive symptoms and brain structure
Source: Dev Cogn Neurosci. 2024 Sep 13;69:101449. doi: 10.1016/j.dcn.2024.101449 (PMC11439534; doi:10.1016/j.dcn.2024.101449)
Supplement: Supplementary file 1 — Supplementary material [file mmc1.docx]

**Supporting Material**

**Supplementary Materials**

**Methods**

**Description of study numbers**

Pregnant women resident in Avon, UK with expected dates of delivery between 1st April 1991 and 31st December 1992 were invited to take part in the study. 20,248 pregnancies have been identified as being eligible and the initial number of pregnancies enrolled was 14,541. Of the initial pregnancies, there was a total of 14,676 foetuses, resulting in 14,062 live births and 13,988 children who were alive at 1 year of age. When the oldest children were approximately 7 years of age, an attempt was made to bolster the initial sample with eligible cases who had failed to join the study originally. As a result, when considering variables collected from the age of seven onwards (and potentially abstracted from obstetric notes) there are data available for more than the 14,541 pregnancies mentioned above: The number of new pregnancies not in the initial sample (known as Phase I enrolment) that are currently represented in the released data and reflecting enrolment status at the age of 24 is 906, resulting in an additional 913 children being enrolled (456, 262 and 195 recruited during Phases II, III and IV respectively). The phases of enrolment are described in more detail in the cohort profile paper and its update (see footnote 5 below). The total sample size for analyses using any data collected after the age of seven is therefore 15,447 pregnancies, resulting in 15,658 foetuses. Of these 14,901 children were alive at 1 year of age.

Study data were collected and managed using REDCap electronic data capture tools hosted at the University of Bristol (Harris et al., 2019). REDCap (Research Electronic Data Capture) is a secure, web-based software platform designed to support data capture for research studies.

Please note that the study website contains details of all the data that is available through a fully searchable data dictionary and variable search tool" and reference the following webpage: <http://www.bristol.ac.uk/alspac/researchers/our-data/>

**Ethics**

Ethical approval for the study was obtained from the ALSPAC Ethics and Law Committee and the Local Research Ethics Committees.

Informed consent for the use of data collected via questionnaires and clinics was obtained from participants following the recommendations of the ALSPAC Ethics and Law Committee at the time.

**MRI subsample**

A subset of ALSPAC children were between the ages 18 and 24 years invited for an MRI scan in one of three studies: i) The ALSPAC testosterone study, ii) the ALSPAC Psychotic Experiences study, and iii) the ALSPAC Schizophrenia recall-by-genotype study. Scanning protocols were harmonized across the three ALSPAC sub-studies and neuroimaging data were acquired at Cardiff University Brain Research Imaging Centre (CUBRIC) on the same 3 Tesla General Electric HDx (GE Medical Systems) using an 8-channel head coil. For more details see Sharp et al. (2020). It is important to highlight that the three subsamples possess distinct characteristics; i) the ALSPAC testosterone study consists of males only (n=513), ii) the ALSPAC Psychotic Experiences study recruited individuals who at age 17-18 scored at least one definite or suspected psychotic experience using the psychotic-like symptoms semi-structured interview (PLIKS, (Horwood et al., 2008)) (n=126), in addition to healthy controls (n=126), and iii) the ALSPAC Schizophrenia recall-by-genotype study included individuals who were selected on basis of low (n=98) or high (n=98) genetic risk for schizophrenia. We will perform analyses with all participants but run sensitivity analyses after excluding individuals with psychotic experiences or high genetic risk for schizophrenia.

**Supplementary Figure 1***Initial data availability*


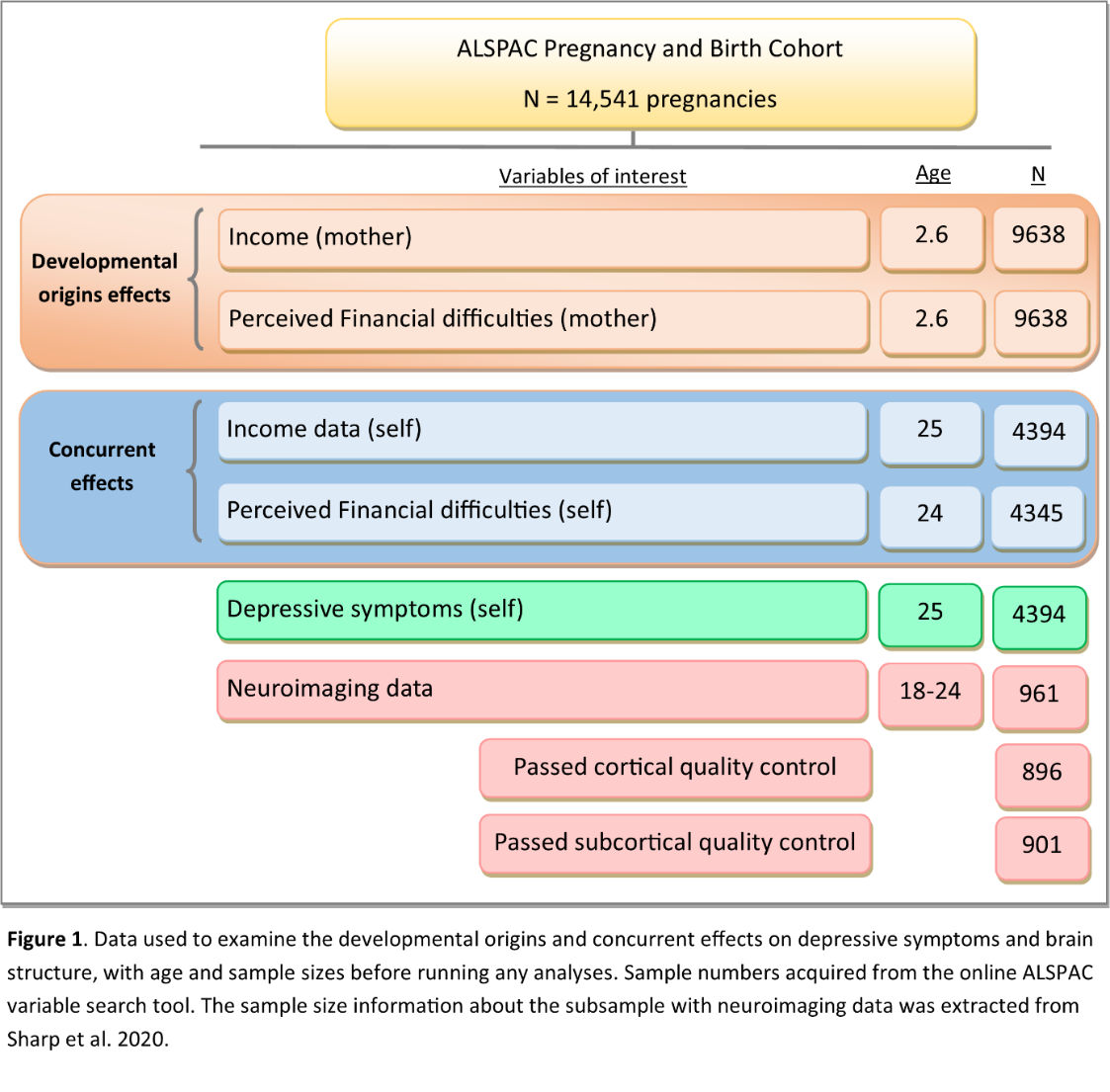


In some case, participants were randomly chosen from the dataset, for example a random choice of a twin from a twin pair, or a random choice of MRI data from the same individual who had MRI data from two time points of equal quality. In such cases, we ensured reproducibility of this and other subsequent randomized processes by setting a seed in R using the set.seed() function. The seed acts as a starting point for the sequence of random numbers generated by R, which means that the same seed will produce the same sequence of random numbers each time. To reduce dependency in the data, we randomly removed a twin from each pair of twins where the MRI data quality was equal. In one case, we removed a twin whose MRI data quality was poorer and retained the twin that had better quality of MRI data.

**Measures**

Maternal depressive symptoms at 21-months postnatally were assessed using the Edinburgh Postnatal Depression Scale (EPDS; Levis et al., 2020). Data distributions is presented in Supplementary figure 2.

**Supplementary Figure 2***Histogram of scores on the Edinburgh Postnatal Depression Scale (EPDS) collected at 21-months postnatally.*


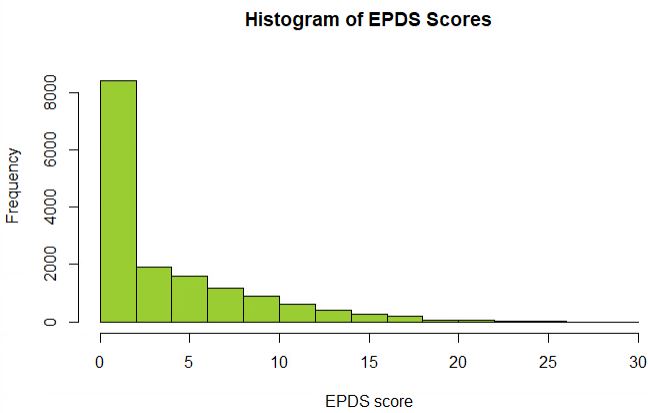


**Results**

For transparency, we included results of the analyses when only individuals with socioeconomic disadvantaged form both time points were included in the analyses, as preregistered. The results were very similar. Supplementary table 1 below shows sample sized when the strict inclusion criterion was used.

**Supplementary Table 1***Sample size numbers for the different analyses using the preregistered stringent inclusion criteria*

| Outcome  Range (mean) | | | Predictor | N | Sex  (% F) | Age in months  Range (mean) |
| --- | --- | --- | --- | --- | --- | --- |
| Mental Health | Depressive symptoms | 0-26(6.62) | Income | 2679 | 65.7 | 296-324 (308.9) |
|  |  | 0-26(6.69) | PDF | 2707 | 66.9 | 296-325 (308.8) |
| Cortical | SA | 129421-222636 (173057) | Income | 388 | 36.6 | 216-292(233) |
|  |  | 1294421-222636(173337) | PFD | 496 | 33.9 | 216-291(245) |
|  | CT | 4.8-5.7(5.31) | Income | 388 | 36.6 | 216-292(233) |
|  |  | 4.8-5.7(5.3) | PFD | 496 | 33.9 | 216-291(245) |
|  | ICV | 1128680-2158650 (1676241) | Income | 388 | 36.6 | 216-292(233) |
|  |  | 1128680-2158650(1678992) | PFD | 496 | 33.9 | 216-291(245) |
| Subcortical | Hippocampus | 6759-11972(9117) | Income | 396 | 35.1 | 216-292(233) |
|  |  | 6413-11972(9127) | PFD | 505 | 33.7 | 216-291(245) |
|  | Amygdala | 2373-4457(3333) | Income | 396 | 35.1 | 216-292(233) |
|  |  | 2373-4457(3334) | PFD | 505 | 33.7 | 216-291(245) |
|  | Striatum | 10920-21866(16898) | Income | 396 | 35.1 | 216-292(233) |
|  |  | 10920-21866(16927) | PFD | 505 | 33.7 | 216-291(245) |

*Notes*: N = sample size, F = females, PDF = perceived financial difficulties, SA = surface area (in mm^2^),
CT = cortical thickness (in mm), ICV = intracranial volume (in mm^3^)
**Supplementary table 1** shows sample sizes and other demographics information had the original strict inclusion criterion been used.

**Associations between socioeconomic disadvantage and depressive symptoms**

**Supplementary Table 2**

| Depression regressed on Income childhood + Age + Sex | | | | |
| --- | --- | --- | --- | --- |
|  | Standardized estimate | SE | t | Uncorrected p |
| Income childhood | -0.080 | 0.018 | -4587 | 4.71e-06*** |
| Age | -0.003 | 0.018 | -0.196 | 0.845 |
| Sex | 0.336 | 0.040 | 8.452 | < 2e-16*** |
| *Note.* ´***´ = 0, ´**´ = 0.001, ´*´ = 0.01, ´.´ = 0.05 | | | | |

**Supplementary Table 3**

| Depression regressed on Concurrent income + Age + Sex | | | | |
| --- | --- | --- | --- | --- |
|  | Standardized estimate | SE | t | Uncorrected p |
| Concurrent income | -0.154 | 0.018 | -8.508 | < 2e-16*** |
| Age | 0.005 | 0.018 | 0.300 | 0.764 |
| Sex | 0.301 | 0.040 | 7.573 | 4.98e-14 *** |
| *Note.* ´***´ = 0, ´**´ = 0.001, ´*´ = 0.01, ´.´ = 0.05 | | | | |

**Supplementary Table 4**

| Depression regressed on Concurrent income + Age + Sex + Childhood income | | | | |
| --- | --- | --- | --- | --- |
|  | Standardized estimate | SE | t | Uncorrected p |
| Concurrent income | -0.144 | 0.018 | -7.840 | < 2e-16 *** |
| Age | 0.002 | 0.018 | 0.114 | 0.910 |
| Sex | 0.299 | 0.040 | 7.551 | 5.89e-14 *** |
| Childhood Income | -0.055 | 0.017 | -3.131 | 0.00176 ** |
| *Note.* ´***´ = 0, ´**´ = 0.001, ´*´ = 0.01, ´.´ = 0.05 | | | | |

**Supplementary Table 5**

| Depression regressed on Concurrent income + Age + Sex + Childhood Income + Maternal depression | | | | |
| --- | --- | --- | --- | --- |
|  | Standardized estimate | SE | t | Uncorrected p |
| Concurrent income | -0.141 | 0.019 | -7.484 | 9.92e-14*** |
| Age | 0.005 | 0.018 | 0.253 | 0.800 |
| Sex | 0.300 | 0.040 | 7.439 | 1.38e-13*** |
| Childhood income | -0.032 | 0.018 | -1.775 | 0.076. |
| Maternal depression | -0.083 | 0.017 | -4.854 | 1.28e-06*** |
| *Note.* ´***´ = 0, ´**´ = 0.001, ´*´ = 0.01, ´.´ = 0.05 | | | | |

**Supplementary Table 6**

| Depression regressed on Concurrent income + Age + Sex + Childhood Income + Ethnicity | | | | |
| --- | --- | --- | --- | --- |
|  | Standardized estimate | SE | t | Uncorrected p |
| Concurrent income | -0.143 | 0.019 | -7.680 | 2.24e-14*** |
| Age | -0.002 | 0.018 | -0.101 | 0.920 |
| Sex | 0.286 | 0.040 | 7.160 | 1.04e-12*** |
| Childhood income | -0.057 | 0.018 | -3.150 | 0.002** |
| Ethnicity | 0.203 | 0.094 | 2.155 | 0.031* |
| *Note.* ´***´ = 0, ´**´ = 0.001, ´*´ = 0.01, ´.´ = 0.05 | | | | |

**Supplementary Table 7**

| Depression regressed on Childhood income + Age + Sex + Ethnicity | | | | |
| --- | --- | --- | --- | --- |
|  | Standardized estimate | SE | t | Uncorrected p |
| Childhood income | -0.082 | 0.018 | -4.579 | 4.89e-06*** |
| Age | -0.007 | 0.018 | -0.384 | 0.701 |
| Sex | 0.311 | 0.040 | 8.024 | 1.53e-15*** |
| Ethnicity | 0.200 | 0.095 | 2.092 | 0.0365 * |
| *Note.* ´***´ = 0, ´**´ = 0.001, ´*´ = 0.01, ´.´ = 0.05 | | | | |

**Supplementary Table 8**

| Depression regressed on Concurrent income + Age + Sex + Ethnicity | | | | |
| --- | --- | --- | --- | --- |
|  | Standardized estimate | SE | t | Uncorrected p |
| Concurrent income | -0.153 | 0.018 | -8.346 | <2e-16*** |
| Age | 0.002 | 0.018 | 0.087 | 0.9309 |
| Sex | 0.287 | 0.040 | 7.162 | 1.03e-12*** |
| Ethnicity | 0.214 | 0.095 | 2.266 | 0.0235* |
| *Note.* ´***´ = 0, ´**´ = 0.001, ´*´ = 0.01, ´.´ = 0.05 | | | | |

**Supplementary Table 9**

| Depression regressed on Concurrent income + Age + Sex + Maternal depression | | | | |
| --- | --- | --- | --- | --- |
|  | Standardized estimate | SE | t | Uncorrected p |
| Concurrent income | -0.147 | 0.019 | -7.867 | 5.36e-15*** |
| Age | 0.007 | 0.018 | 0.368 | 0.713 |
| Sex | 0.301 | 0.040 | 7.445 | 1.32e-13*** |
| Maternal depression | -0.087 | 0.017 | -5.149 | 2.82e-07*** |
| *Note.* ´***´ = 0, ´**´ = 0.001, ´*´ = 0.01, ´.´ = 0.05 | | | | |

**Supplementary Table 10**

| Depression regressed on Childhood income + Age + Sex + Maternal depression | | | | |
| --- | --- | --- | --- | --- |
|  | Standardized estimate | SE | t | Uncorrected p |
| Childhood income | -0.055 | 0.018 | -3.027 | 0.00249** |
| Age | -0.002 | 0.018 | -0.105 | 0.916 |
| Sex | 0.336 | 0.041 | 8.295 | <2e-16*** |
| Maternal depression | -0.090 | 0.017 | -5.240 | 1.74e07*** |
| *Note.* ´***´ = 0, ´**´ = 0.001, ´*´ = 0.01, ´.´ = 0.05 | | | | |

**Supplementary Figure 3**

*Timing-specific associations between childhood and concurrent income on adult depression*


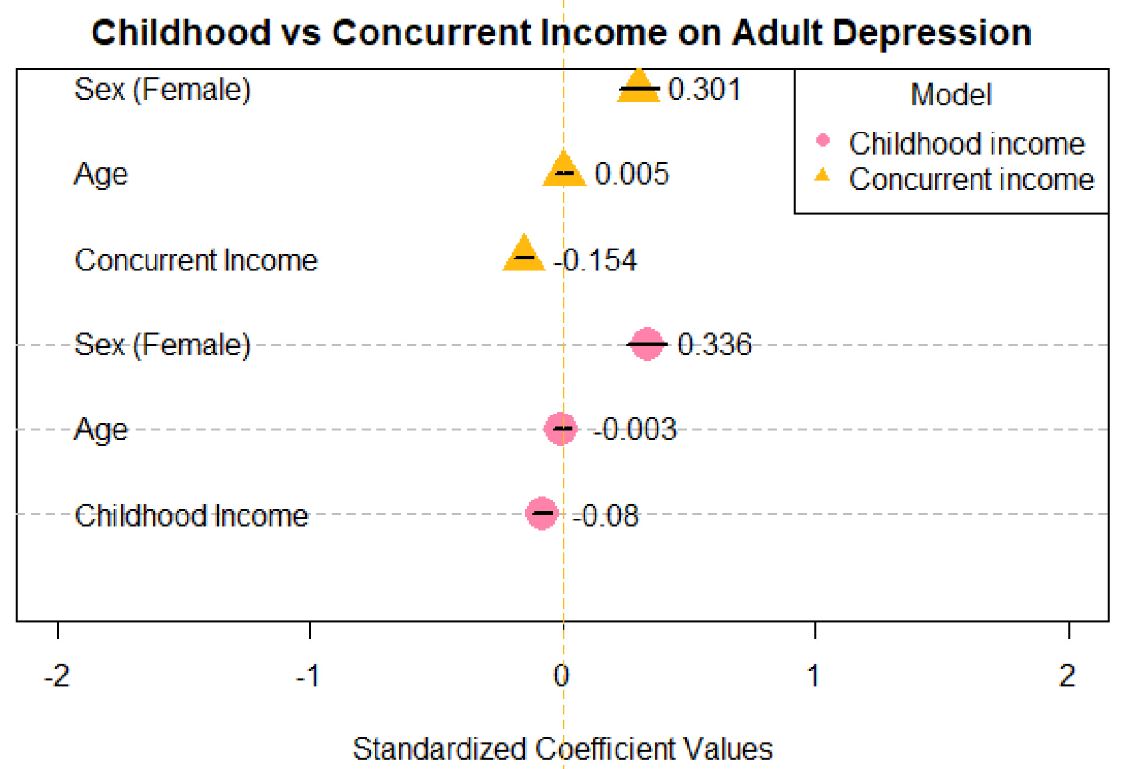


*Note.*  standardized beta coefficients from two generalized linear models: depressive symptoms regressed on concurrent income + age + sex (yellow triangles); depressive symptoms childhood income + age + sex (pink circles).

**Supplementary Figure 4**

*Independent timing-specific associations between childhood and concurrent income on adult depression*


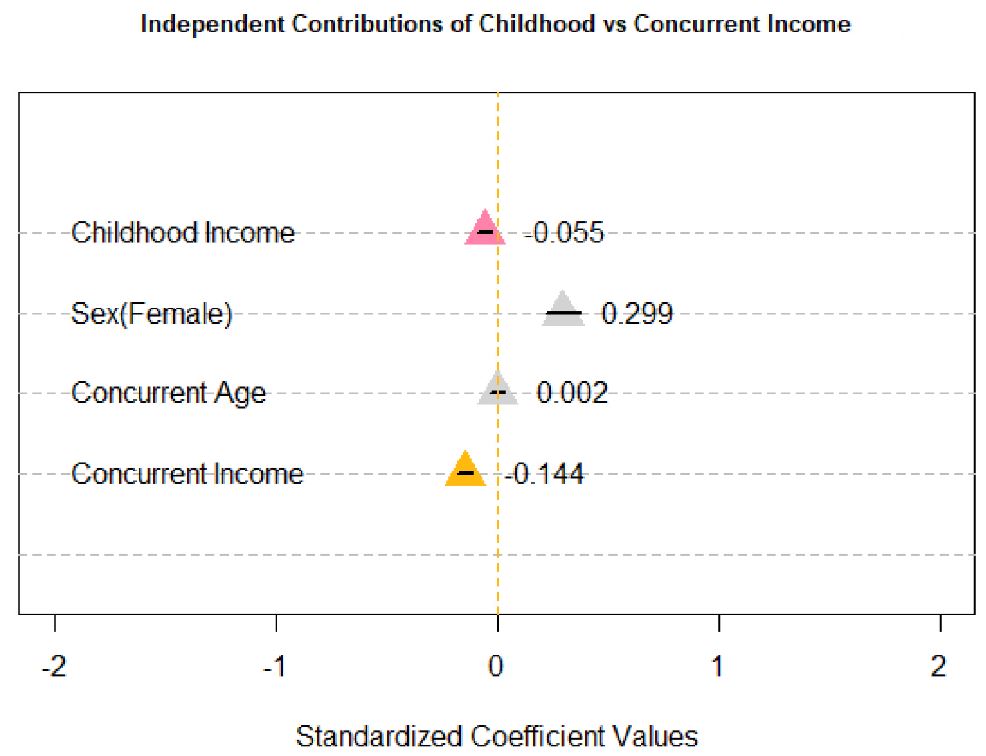


*Note.* Variance of depressive symptoms childhood income explained above and beyond concurrent income (and vice versa)

**Supplementary Table 11**

| Depression regressed on Childhood PFD + Age + Sex | | | | |
| --- | --- | --- | --- | --- |
|  | Standardized estimate | SE | t | Uncorrected p |
| Childhood PFD | 0.064 | 0.017 | 3.836 | 0.0001*** |
| Age | 0.006 | 0.018 | 0.326 | 0.744 |
| Sex | 0.306 | 0.041 | 7.533 | 6.71e-14 |
| *Note.* ´***´ = 0, ´**´ = 0.001, ´*´ = 0.01, ´.´ = 0.05 | | | | |

**Supplementary Table 12**

| Depression regressed on Concurrent PFD + Age + Sex | | | | |
| --- | --- | --- | --- | --- |
|  | Standardized estimate | SE | t | Uncorrected p |
| Concurrent PFD | 0.174 | 0.014 | 12.154 | < 2e-16*** |
| Age | 0.003 | 0.018 | 0.181 | 0.856 |
| Sex | 0.300 | 0.040 | 7.477 | 1.02e-13*** |
| *Note.* ´***´ = 0, ´**´ = 0.001, ´*´ = 0.01, ´.´ = 0.05 | | | | |

**Supplementary Table 13**

| Depression regressed on Concurrent PFD + Age + Sex + Childhood PFD | | | | |
| --- | --- | --- | --- | --- |
|  | Standardized estimate | SE | t | Uncorrected p |
| Concurrent PFD | 0.168 | 0.145 | 11.605 | <2e-16*** |
| Age | 0.002 | 0.0176 | 0.119 | 0.905 |
| Sex | 0.294 | 0.040 | 7.379 | 2.11e-13*** |
| Childhood PFD | 0.036 | 0.017 | 2.136 | 0.033* |
| *Note.* ´***´ = 0, ´**´ = 0.001, ´*´ = 0.01, ´.´ = 0.05 | | | | |

**Supplementary Table 14**

| Depression regressed on Childhood PFD + Age + Sex + Ethnicity | | | | |
| --- | --- | --- | --- | --- |
|  | Standardized estimate | SE | t | Uncorrected p |
| Childhood PFD | 0.059 | 0.017 | 3.487 | 0.001*** |
| Age | 0.003 | 0.018 | 0.139 | 0.890 |
| Sex | 0.288 | 0.041 | 7.071 | 1.96e-12*** |
| Ethnicity | 0.196 | 0.095 | 2.067 | 0.039* |
| *Note.* ´***´ = 0, ´**´ = 0.001, ´*´ = 0.01, ´.´ = 0.05 | | | | |

**Supplementary Table 15**

| Depression regressed on Concurrent PFD + Age + Sex + Ethnicity | | | | |
| --- | --- | --- | --- | --- |
|  | Standardized estimate | SE | t | Uncorrected p |
| Concurrent PFD | 0.179 | 0.014 | 12.330 | < 2e-16*** |
| Age | -0.001 | 0.018 | -0.044 | 0.965 |
| Sex | 0.280 | 0.040 | 7.038 | 2.47e-12*** |
| Ethnicity | 0.218 | 0.093 | 2.347 | 0.019* |
| *Note.* ´***´ = 0, ´**´ = 0.001, ´*´ = 0.01, ´.´ = 0.05 | | | | |

**Supplementary Table 16**

| Depression regressed on Concurrent PFD + Age + Sex + Childhood PFD + Ethnicity | | | | |
| --- | --- | --- | --- | --- |
|  | Standardized estimate | SE | t | Uncorrected p |
| Concurrent PFD | 0.174 | 0.015 | 11.855 | < 2e-16*** |
| Age | -0.002 | 0.018 | -0.110 | 0.924 |
| Sex | 0.277 | 0.040 | 6.952 | 4.52e-12*** |
| Childhood PFD | 0.032 | 0.017 | 1.863 | 0.063. |
| Ethnicity | 0.215 | 0.092 | 2.323 | 0.0202* |
| *Note.* ´***´ = 0, ´**´ = 0.001, ´*´ = 0.01, ´.´ = 0.05 | | | | |

**Supplementary Table 17**

| Depression regressed on Childhood PFD + Age + Sex + Maternal depression | | | | |
| --- | --- | --- | --- | --- |
|  | Standardized estimate | SE | t | Uncorrected p |
| Childhood PFD | 0.038 | 0.018 | 2.151 | 0.0316* |
| Age | 0.011 | 0.018 | 0.626 | 0.532 |
| Sex | 0.300 | 0.041 | 7.272 | 4.67e-13*** |
| Maternal depression | -0.109 | 0.018 | -6.096 | 1.25e-09*** |
| *Note*. ´***´ = 0, ´**´ = 0.001, ´*´ = 0.01, ´.´ = 0.05 | | | | |

**Supplementary Table 18**

| Depression regressed on Concurrent PFD + Age + Sex + Maternal depression | | | | |
| --- | --- | --- | --- | --- |
|  | Standardized estimate | SE | t | Uncorrected p |
| Concurrent PFD | 0.018 | 0.015 | 11.841 | < 2e-16*** |
| Age | 0.010 | 0.018 | 0.542 | 0.588 |
| Sex | 0.290 | 0.040 | 7.185 | 8.77e-13*** |
| Maternal depression | -0.107 | 0.017 | -6.289 | 3.75e-10*** |
| *Note.* ´***´ = 0, ´**´ = 0.001, ´*´ = 0.01, ´.´ = 0.05 | | | | |

**Supplementary Table 19**

| Depression regressed on Concurrent PFD + Age + Sex + Childhood PFD + Maternal depression | | | | |
| --- | --- | --- | --- | --- |
|  | Standardized estimate | SE | t | Uncorrected p |
| Concurrent PFD | 0.171 | 0.015 | 11.586 | < 2e-16*** |
| Age | 0.009 | 0.018 | 0.514 | 0.607 |
| Sex | 0.288 | 0.040 | 7.145 | 1.17e-12*** |
| Childhood PFD | 0.011 | 0.017 | 0.615 | 0.538 |
| Maternal depression | -0.104 | 0.017 | -5.984 | 2.48e-09*** |
| *Note*. ´***´ = 0, ´**´ = 0.001, ´*´ = 0.01, ´.´ = 0.05 | | | | |

**Supplementary Figure 5**

*Timing-specific associations between childhood and concurrent perceived financial difficulties on adult depression*


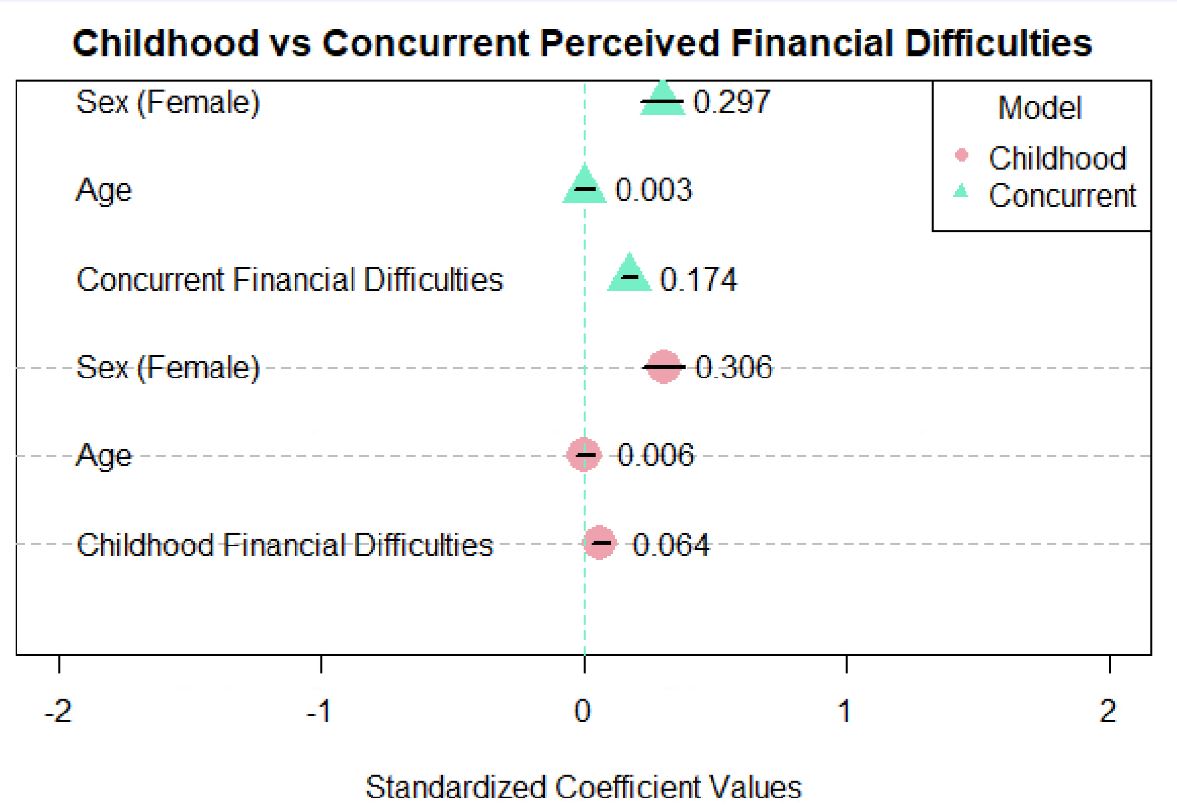


*Note.* standardized beta coefficients from two generalized linear models: depressive symptoms regressed on concurrent PDS + age + sex (green triangles); depressive symptoms regressed onchildhood PFD+ age + sex (pink circles).

**Supplementary Figure 6**

*Independent timing-specific associations between childhood and concurrent perceived financial difficulties on adult depression*


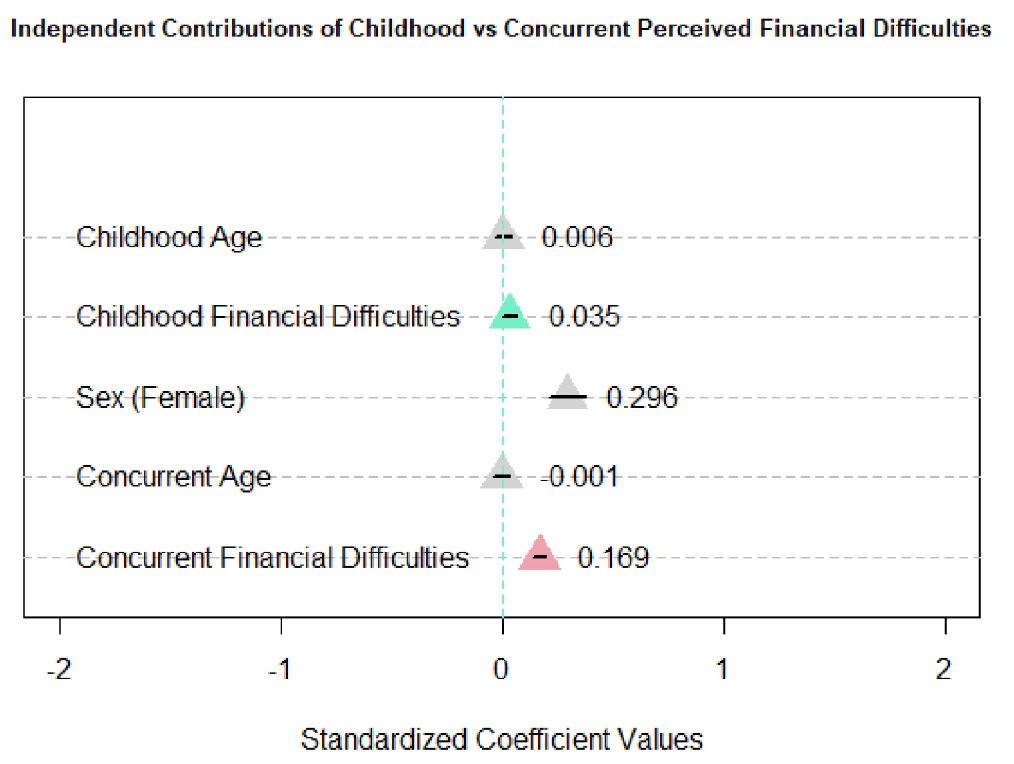


*Note.* Variance of depressive symptoms childhood PFD explained above and beyond concurrent PFD (and vice versa)

**Associations between socioeconomic disadvantage and brain structure**

The tables below show results for analyses with ICV and total surface area as outcomes. The tables show standardized beta values.

**Supplementary Table 20**

| ICV regressed on Childhood income, Age and Sex | | | | |
| --- | --- | --- | --- | --- |
|  | Standardized estimate | SE | t | Uncorrected p |
| Childhood income | 0.117 | 0.040 | 2.893 | 0.00404 |
| Age | -0.049 | 0.039 | -1.263 | 0.20731 |
| Sex | -1.31521 | 0.08309 | -15.828 | <2e-16 |
| *Note.* ´***´ = 0, ´**´ = 0.001, ´*´ = 0.01, ´.´ = 0.05 | | | | |

**Supplementary Table 21**

| SA regressed on Childhood income, Age and Sex | | | | |
| --- | --- | --- | --- | --- |
|  | Standardized estimate | SE | t | Uncorrected p |
| Childhood income | 0.086 | 0.043 | 2.013 | 0.0448 |
| Age | -0.056 | 0.041 | -1.342 | 01805 |
| Sex | -1.213 | 0.088 | -13.808 | <2e-16 |
| *Note.* ´***´ = 0, ´**´ = 0.001, ´*´ = 0.01, ´.´ = 0.05 | | | | |

In the analyses where individuals were required to have income and PFD data from both time points, childhood income was related significantly to total SA and ICV, however, the associations did not survive correction for multiple comparison. It was likely due to the loss of power, as using this stringent inclusion criterium meant losing about one third of the sample with MRI data.

**Exploratory vertex-wise analyses**

**Supplementary Figure 7***Associations between childhood income and surface area while controlling for age and sex*


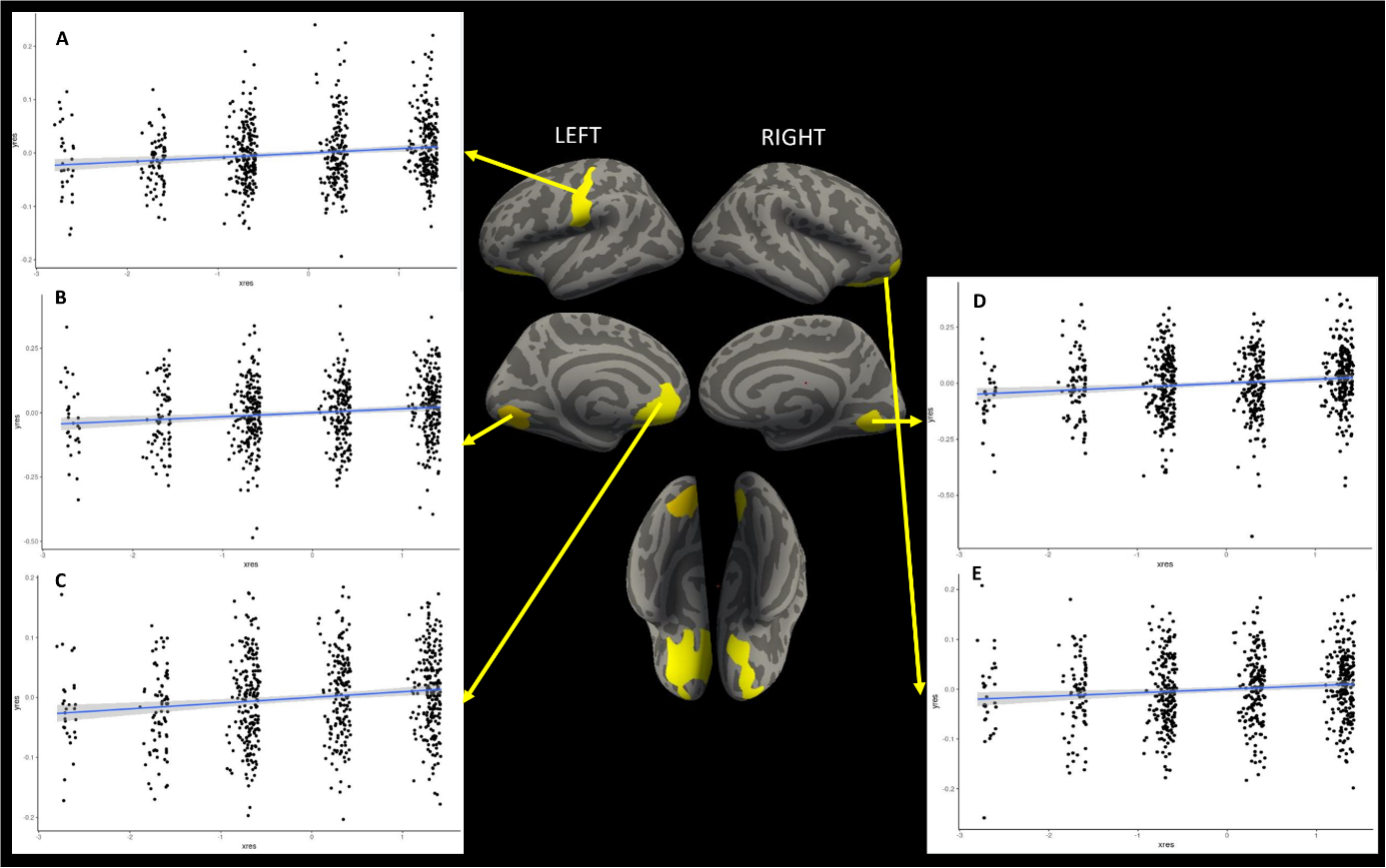

*Note*. Scatterplots show residual surface area and income in A) postcentral gyrus, B) lingual gyrus, C) medial orbitofrontal cortices, D) lingual gyrus and E) lateral orbitofrontal cortices.

**Supplementary Figure 8**

*Associations between childhood income and surface area while controlling for age and sex, using a more liberal threshold of 0.05*


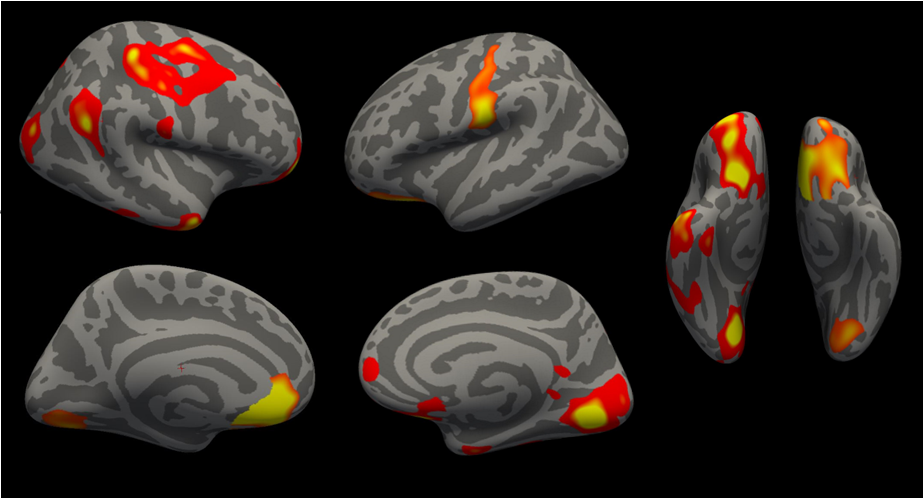

*Note.* Yellow-red colors indicate positive associations

**Post-hoc analyses:**

Analyses with subjective and objective measures in the same model

When repeating analyses with childhood perceived financial difficulties as additional covariate, childhood income was still negatively associated with adult depressive symptoms β = -0.10, *p* < 0.001, CI [-0.13,-0.06]. Similarly, concurrent income was still negatively associated with adult depressive symptoms, above and beyond concurrent perceived financial difficulties: β = -0.14, *p* < 0.001, CI [-0.18,-0.11]. Early childhood perceived financial difficulties were still positively associated with adult depressive symptoms even when we included childhood income as a covariate, although the effect was weaker, β = -0.04, *p* = 0.033, CI [0.00 - 0.07]. Concurrent perceived financial difficulties were positively associated with depressive symptoms in emerging adulthood, above and beyond concurrent income: β = 0.14, *p* < 0.001, CI [0.11 - 0.17]. Comparing the beta coefficients in each model, again income appeared to be a stronger predictor. The only exception were concurrent perceived financial difficulties that appeared to have similar beta values: β = 0.14, *p* < 0.001, CI [0.11 - 0.17] as concurrent income: β = -0.14, *p* < 0.001, CI [-0.7, -0.11].

Analyses including both childhood and concurrent income in predicting brain structure.

**Supplementary Table 22**

| Total surface area regressed on childhood income, age at MRI and sex | | | | |
| --- | --- | --- | --- | --- |
|  | β | *Std.Error* | *T value* | *p* |
| Intercept | 0.42 | 0.05 | 8.16 | < .001 |
| Childhood income | 0.09 | 0.04 | 2.01 | .044 |
| Age | -0.06 | 0.04 | -1.34 | .181 |
| Sex | -1.21 | 0.09 | -13.8 | < .001 |

**Supplementary Table 23**

| Total surface area regressed on childhood income, age at MRI, sex and concurrent income | | | | |
| --- | --- | --- | --- | --- |
|  | β | *Std.Error* | *t value* | *p* |
| Intercept | 0.42 | 0.05 | 8.16 | < .001 |
| Childhood income | 0.08 | 0.04 | 1.8 | .079 |
| Age | -0.06 | 0.04 | -1.40 | < .001 |
| Sex | -1.21 | 0.09 | -13.8 | < .001 |
| Concurrent income | 0.04 | 0.038 | 1.03 | 0.304 |

**Supplementary Table 24**

| Intercranial volume regressed on childhood income, age at MRI and sex | | | | |
| --- | --- | --- | --- | --- |
|  | β | *Std.Error* | *T value* | *p* |
| Intercept | 0.38 | 0.04 | 10.1 | < .001 |
| Childhood income | 0.10 | 0.03 | 3.04 | 0.003 |
| Age | -0.04 | 0.03 | -1.43 | 0.155 |
| Sex | -1.36 | 0.07 | -19.9 | < .001 |

**Supplementary Table 25**

| Intercranial volume regressed on childhood income, age at MRI, sex and concurrent income | | | | |
| --- | --- | --- | --- | --- |
|  | β | *Std.Error* | *T value* | *p* |
| Intercept | 0.40 | 0.05 | 7.34 | < .001 |
| Childhood income | 0.12 | 0.05 | 2.47 | 0.014 |
| Age | -0.05 | 0.04 | -1.29 | 0.198 |
| Sex | -1.40 | 0.09 | -15.65 | < .001 |
| Concurrent income | 0.06 | 0.04 | 1.46 | 0.145 |
